# Progress in Surgery

**Published:** 1898-09-24

**Authors:** 


### Progress in Surgery.

### GENERAL SURGERY.

*(Concluded from page 430.)*

**Lower Extremity.**—Mr. W. A. Lane[22] performed a somewhat novel operation on a case of fracture of the neck of the femur, which had taken place in a boy aged 14 some months previously. Besides the usual signs of this injury, flexion of the hip-joint took place around an axis which ran from behind, forwards and inwards, forming a very much larger angle with the vertical transverse plane than was the case in the normal joint. To approximate the points of attachment of the ilio-femoral ligament, in order to remedy the outward rotation of the thigh, he successfully shortened the ligament by means of loops of strong silver wire passed parallel to its fibres. An illustrated description of severe deformity of the femora in a youth aged 18 years, due to repeated fracture of these bones, is given by Dr. D. Brough.[23] The patient displayed peculiar liability to fracture, although the family history showed no hereditary tendency to fragilitas ossium. Altogether he had sustained nine fractures, five being in the left femur, three in the right femur, and one in the right clavicle. Two of the fractures in the left femur were situated in the middle, and three at the junction of the upper and middle thirds. In the right femur, one occurred in the middle, one at the junction of the upper and middle thirds, while the site of one, occurring in infancy, was unknown. On each occasion the accident was very slight, and the resulting fractures were quite disproportionate to the nature of the fall. Dr. Albert Bouffleur[24] records an instance of simple epiphysial fracture of the lower end of the femur, in which the condyloid fragment was displaced forwards and mistaken for a dislocation of the tibia. Although two weeks had elapsed since the accident the fragments were, under an anæsthetic, completely reduced by strong extension downwards and backwards, and by firm manipulation. The limb was then placed on a double inclined plane and extension applied. A very large proportion of cases of fracture of the patella, Charles B. Ball[25] remarks, undoubtedly recover with a sufficiently useful limb after treatment by ordinary methods, even where the union is not by bone; and when these methods fail a secondary operation can be undertaken with every prospect of success. As regards the latter, he recommends the usual horse-shoe flap, and passes a separate piece of fine steel wire (made of eight strands) round the upper and the lower fragments, and twists them together at each side of the bone rather than encircle the patella with a single piece and twist the ends at one side only. By this means the right degree of tension can more readily be obtained, and the adjustment made more perfect. Mr. Ball relates a case in which he adopted this method successfully, skiagraphs being given of the fracture before and after operation. After having performed most of the modern operations for the recent treatment of transverse fracture of the patella, T. Vincent Jackson[26] gives, as a result of his experience, that if an operation is desirable in a recent case, the easiest, the most rapidly performed, and the most quickly recovered from is Mr. Barker's modification of Kocher's method. It is necessary, he says, to be scrupulously careful in every detail, and a qualitative examination of the urine should always be made previously. He uses strong silk for encircling the patella, never wire as Mr. Barker does. Where the open operation for fractured patella is not admissible, an alternative method of apposing the fragments by external mechanical means is attained by the apparatus recently figured and described by Heather Biggs.[27] He believes that the feature of this particular treatment is that not only are the segments safely held in apposition, but the reunited bone is submitted by controllable graduations to movement and to strain, and the new tissue of union is thereby provoked into attaining perfect strength. He has notes of nearly three hundred cases satisfactorily dealt with by this method, and he adds that with some modifications of detail it is equally applicable to ruptured quadriceps and to ruptured ligamentum patellæ. Among twenty-two consecutive arthrotomies of the knee, Dr. John O'Conor[28] has treated five cases of traumatic hæmarthrosis of the knee successfully; while a fifth case of old hæmarthrosis with united vertical fracture of the patella was treated by this method of free incision, and by division of fibrous bands behind the patella with scissors. It was the case of a sailor, aged 30, who had fractured his kneecap three months previously, and at the time of operation any attempt at passive movement caused intense pain. He was discharged from hospital on the fortieth day, and walked without limp or impaired function. A month later he undertook stevedore's work. Mr. Stanley Boyd[29] alludes to a case of ununited fracture of the tibia in a child aged three years. The fracture was situated between the lower fourth and upper three-fourths of the bone, and the ends of the fragments were only united by fibrous material thirteen months after the accident. After twelve or thirteen months' rest to the limb, and massage, he proposed to operate, freshen the ends, and unite them by by means of a central peg of ivory, or by wire or a screw.

[22] Practitioner, May, 1898, p. 515. [23] Lancet, May 7, 1898, p. 1,249. [24] Clinical Review, Mar., 1898, p. 333. [25] Practitioner, May, 1898, p. 483. [26] Birmingham Med. Review, Feb., 1898, p. 83. [27] Lancet, Aug. 20, 1898, p. 479, [28] Glasgow Med. Jour., May, 1898, p. 353, and Amer. Jour. Med. Sci., July, 1898, p. 105. [29] Clinical Jour., April 13, 1898, p, 444.

